# Exogenously applied gibberellic acid and benzylamine modulate growth and chemical constituents of dwarf schefflera: a stepwise regression analysis

**DOI:** 10.1038/s41598-024-57985-0

**Published:** 2024-04-03

**Authors:** Ali Salehi Sardoei, Mojtaba Tahmasebi, Fatemeh Bovand, Mansour Ghorbanpour

**Affiliations:** 1 Crop and Horticultural Science Research Department, South Kerman Agricultural and Natural Resources Research and Education Center, AREEO, Jiroft, Iran; 2 Department of Landscape Architecture, University of Florida, Gainesville, FL USA; 3 Department of Agronomy and Plant Breeding, Islamic Azad University, Arak, Iran; 4 Department of Medicinal Plants, Faculty of Agriculture and Natural Resources, Arak University, Arak, 38156-8-8349 Iran

**Keywords:** Dwarf schefflera, Leaf number, Path test, Plant growth regulators, Stepwise regression, Physiology, Plant sciences

## Abstract

Ornamental foliage plants that have a dense appearance are highly valued. One way to achieve this is by using plant growth regulators as a tool for plant growth management. In a greenhouse with a mist irrigation system, a study was conducted on dwarf schefflera, an ornamental foliage plant, which was exposed to foliar application of gibberellic acid and benzyladenine hormones. The hormones were sprayed on dwarf schefflera leaves at 0, 100, and 200 mg/l concentrations, at 15-day intervals in three stages. The experiment was conducted as a factorial based on a completely randomized design, with four replicates. The combination of gibberellic acid and benzyladenine at 200 mg/l concentration had a significant effect on leaf number, leaf area, and plant height. The treatment also resulted in the highest content of photosynthetic pigments. Furthermore, the highest soluble carbohydrate to reducing sugars ratio was observed in treatments of 100 and 200 mg/l benzyladenine, and 200 mg/l gibberellic acid + benzyladenine. Stepwise regression analysis showed that root volume was the first variable to enter the model, explaining 44% of variations. The next variable was root fresh weight, and the two-variable model explained 63% of variations in leaf number. The greatest positive effect on leaf number was related to root fresh weight (0.43), which had a positive correlation with leaf number (0.47). The results showed that 200 mg/l concentration of gibberellic acid and benzyladenine significantly improved morphological growth, chlorophyll and carotenoid synthesis, and reducing sugar and soluble carbohydrate contents in dwarf schefflera.

## Introduction

Dwarf schefflera (*Scheffleraarboricola* (Hayata) Merr.) is a type of evergreen ornamental plant from the Araliacea family that is native to China and Taiwan^1^. This plant is often grown as a pot plant indoors, but only one plant can grow in such conditions. Its leaves have 5 to 16 leaflets, each measuring 10–20 cm in length^2^. Dwarf schefflera is sold in large quantities each year, but modern horticultural techniques are rarely used. Therefore, the use of plant growth regulators as an effective management tool to improve the growth and sustainable production of horticultural products requires more attention. Nowadays, the use of plant growth regulators has increased significantly^3–5^. Gibberellic acid is a plant growth regulator that can improve plant yield^6^. One of its known effects is the stimulation of vegetative growth, including stem and root elongation and leaf area expansion^7^. The most significant effect of gibberellins is the increase in stem height through internode elongation. Foliar spraying of gibberellins on Dwarf plants that are unable to produce gibberellins leads to stem elongation and an increase in plant height^8^. Foliar spraying of gibberellic acid at a concentration of 500 mg/l on laceleaf led to an increase in plant height, leaf number, and leaf width and length^9^. Growth stimulation by gibberellins has been reported in several broad-leaf species^10^. Stem elongation due to foliar spraying of gibberellic acid is also reported in the Scots pine (*Pinussylvestris*) and the white spruce (*Piceaglauca*)^11^.

In a study, the effect of three cytokinin plant growth regulators were investigated on the formation of auxiliary branches in peace lily var. Bent. The experiments were conducted in autumn and spring to examine the seasonal effects. The results showed that kinetin, benzyladenine, and 2-isopentenyladenine did not affect the formation of auxiliary branches. However, in autumn and spring experiments, benzyladenine at 500 ppm resulted in the production of 12.2 and 8.2 auxiliary branches respectively, as compared to 4.9 and 3.9 branches in control plants. The summer treatment was found to be more effective than the winter treatment^12^. In another experiment, peace lily var. Tasson plants were treated with 0, 250, and 500 ppm benzyladenine in 10 cm pots. The results showed that soil treatment significantly increased the number of auxiliary leaves compared to control plants and plants sprayed with benzyladenine. New auxiliary leaves were observed four weeks after treatment and the highest leaf production was seen eight weeks after treatment. The height increase in soil-treated plants 20 weeks after treatment was lower than those which had been^13^. It was reported that benzyladenine at a 20 mg/l concentration significantly increased plant height and leaf number in croton^14^. In calla lily, the application of benzyladenine at a 500 ppm concentration resulted in a greater number of branches, while the lowest value was obtained in the control group^15^. The study aimed to investigate the foliar spraying of gibberellic acid and benzyladenine to improve the growth of dwarf schefflera, a foliar ornamental plant. These plant growth regulators could help commercial growers plan suitable production throughout the year. This issue has not been researched on improvement of growth in dwarf schefflera.

## Materials and methods

### Plant materials

This study was done at the research greenhouse for houseplants of Azad Islamic University, Jiroft Branch, Iran. Homogenous rooted grafts of dwarf schefflera with a height of 25 ± 5 cm were prepared (they were propagated six months before the experiment) and sown in pots. The pots were plastic and black with a diameter of 20 cm and a height of 30 cm^16^.

### Culture conditions

The culturemedium in this study was a mixture of peat moss, humus, washed sand and rice husk in a 1:1:1:1 ratio (v/v)^16^. Alayer of pebbles was placed in the bottom of pots for drainage. The average day and night temperature at the greenhouse in late spring and summer was 32 ± 2 and 28 ± 2 °C, respectively. Relative humidity fluctuated to > 70%. Irrigation was done using a mist system. In average, the plants were irrigated 12 times daily. In autumn and summer, each irrigation lasted for 8 min and the time interval between the irrigations was one hour. Plants were nurtured similarly with a micronutrient solution (Ghoncheh Co., Iran) at 3 ppm concentration four times-i.e. 2, 4, 6 and 8 weeks after sowing- and the plants were irrigated with 100 cc of the solution each time. The nutrient solution contained N at 8 ppm, P at 4 ppm, K at 5 ppm, and microelements of Fe, Pb, Zn, Mn, Mo and B.

### Treatments

Gibberellic acid and benzyladenine plant growth regulators (purchased from Sigma Company) at three levels of 0, 100 and 200 mg/l were prepared and sprayed on plant shoots in three stages with 15-day intervals^17^. Tween 20 (0.1%) (purchased from Sigma Company) was used in the solution to enhance its longevity and uptake. Hormone spraying was done inthe early hours of the morning using a sprayer on shoots and leaves of dwarf schefflera. Control plants were sprayed with distilled water.

### Measured traits

Plant height, stem diameter, leaf area, chlorophyll content, internode number, auxiliary branch length, auxiliary branch number, root volume, root length, leaf, root, stem and total dry and fresh weights, photosynthetic pigment content (chlorophyll a, chlorophyll b, total chlorophyll, carotenoids, total pigments), reducing sugars, and soluble carbohydrates were measured in different treatments.

Chlorophyll content was measured 180 days after spraying using a chlorophyll meter (Spad CL-01) during 9:30–10 AM (due to leaf freshness) in young leaves. Also, leaf area was measured 180 days after spraying. Three leaves from the upper, middle and lower parts of the stem were weighted from each pot. These leaves were then used as stencils on A4 papers, then the obtained patterns were cut. The weight and surface area of a single A4 paper was also measured. Then, the area of the stenciled leaves was calculated by proportioning. Also, root volume was determined using a measuring cylinder. Leaf dry weight, stem dry weight, root dry weight and total dry weight of each sample were measured by drying in an oven at 72 °C for 48 h.

Chlorophyll and carotenoid content was measured using the Lichtenthaler^18^ method. For this purpose, 0.1 g of the fresh leaf was ground in porcelain mortars containing 15 ml of 80%acetone, and after filtering, their absorption was read at 663.2, 646.8 and 470 nm wavelength using a spectrophotometer. The apparatus was calibrated using 80% acetone. The concentration of the photosynthetic pigments was calculated using the following equations:

Chl_a_ = 12.25 A_663.2_—2.79 A_646.8_

Chl_b_ = 21.21 A_646.8_—5.1 A_663.2_

ChlT = chla + chlb.

Car = (1000A_470_ -1.8 Chla—85.02 Chlb)/198.

In which Chl a, Chl b, Chl T and Car are chlorophyll a, chlorophyll b, total chlorophyll and carotenoids, respectively. The results were reported in mg/ ml of plant.

Reducing sugars were measured using the Somogy^19^ method. For this purpose, 0.02 g of the plant shoot was ground with 10 ml of distilled water in a porcelain mortar and then transferred into a small beaker. The beaker was heated until boiling, then its content was filtered using a Whatman paper No. 1 and plant extract was obtained. Two ml of each extract was transferred into a test tube, and 2 ml of copper sulphate solution was added. The tubes were sealed with cotton and heated for 20 min in a water bath at 100 °C. At this stage, Cu^2+^ is converted to Cu_2_O by the reduced aldehyde monosaccharide, and a salmon (terracotta) colour is visible in the bottom of the test tube. After the tubes were cooled, 2 ml of phosphomolybdic acid was added and a blue color became visible. The tubes were shaken intensely until this colour was spread in the tube homogenously. The solution absorption rate was read at 600 nm using a spectrophotometer.

The concentration of reducing sugars was calculated using a standard curve. The concentration of soluble carbohydrates was determined using the Fales^20^ method. For this purpose, 0.1 g of shoot was mixed with 2.5 ml of 80% ethanol at 90 °C for 60 min (two 30-min stages) to extract the soluble carbohydrates. The extracts were then filtered and their alcohol was evaporated. The obtained pellet was solved in 2.5 ml of distilled water. Two hundred ml of each sample was poured into a test tube and 5 ml of anthrone indicator was added. The mixture was placed in a bain- marie for 17 min at 90 °C and its absorption was read at 625 nm after cooling down.

### Statistical analysis

This experiment was carried out as factorial based on a completely randomized design with four replicates. PROC UNIVARIATE procedure was used to investigate the normality of data distribution before the analysis of variance. Statistical analyses commenced with descriptive statistical analysis to comprehend the collected initial data’s quality. Calculations aimed at simplifying and condensing extensive datasets for easier interpretation. Subsequently, more complex analyses were performed. The Duncan’s test, executed using SPSS software (v. 24; IBM Corp., Armonk, NY, USA), was utilized to compute mean squares and experimental errors to determine differences among datasets. The Duncan’s Multiple Range Test (DMRT) at a significance level of (0.05 ≤ p) was employed to identify distinctions between means. The Pearson correlation coefficient (r) was calculated to evaluate the correlation between different parameter pairs using SPSS software (v. 26; IBM Corp., Armonk, NY, USA). Furthermore, linear regression analysis using SPSS software (v. 26) was utilized to predict the first-year variable values based on second-year variable values.On the other hand, a stepwise regression analysis at p < 0.01 was done to determine the key attributes affecting the leaves of dwarf schefflera. Path analysis was done to determine the direct and indirect effects of each attribute in the model (based on the traits which explained the variations the better). All calculations mentioned above (normality of data distribution, simple correlation coefficient, stepwise regression and path analysis) were performed using the SPSS V.26 software.

### Statement on experimental research and field studies on plants

The cultivated plants sampled comply with relevant institutional, national, and international guidelines and domestic legislation of Iran.

## Results

### Descriptive statistics

Descriptive statistics of average, standard deviation, minimum, maximum, range and phenotypic coefficient of variation (CV) for different attributes are summarized in Table 1. Among these statistics, CV makes the comparison of attributes possible due to being dimensionless. Reducing sugars (40.39%), root dry weight (37.32%), root fresh weight (37.30%) carbohydrate to sugars ratio (30.20%) and root volume (30%) had the highest and chlorophyll content (9.88%) and leaf area index (11.77%) had the lowest CV values, respectively. Table 1 shows that total fresh weight had the highest range. However, this trait did not have the highest CV. Therefore, dimensionless indices such as CV should be used to compare the variations of attributes. A high CV indicates a high variation among the treatments for the attribute. In this experiment, the results showed a high variation among the treatments for reducing sugars, root dry weight, root fresh weight, carbohydrate to sugar ratio and root volume traits.

**Table 1:** Descriptive statistics of various parameters in dwarf schefflera.

Traits\Statistic	Mean ± SD	Min	Max	Range	PCV (%)
Leaf chlorophyllIndex	53 ± 5.23	42.35	61.93	19.58	9.88
Internode	45.46 ± 9.55	29.33	71.66	42.33	21
LAI	79.71 ± 9.38	62.55	101.59	39.04	11.77
N. leaf	25 ± 4.64	15	33	18	18.57
Plant height	76.31 ± 10.06	55	96	41	13.19
Diameter stem	0.88 ± 0.16	0.64	1.5	0.86	18.32
Ch. a	18.94 ± 3.43	8.94	24.35	15.41	18.09
Ch. b	7.26 ± 1.73	5.04	11.84	6.8	23.83
a/b Ratio	2.66 ± 0.44	1.68	4.09	2.41	16.41
Ch. total	26.2 ± 4.82	14.24	33	18.76	18.39
Carotenoid	2.9 ± 0.67	1.77	4.97	3.2	23.11
Yield pigment	29.04 ± 4.68	16.5	37.97	21.47	16.11
Reducing sugars	0.29 ± 0.12	0.16	0.76	0.6	40.39
Carbohydrate total	0.65 ± 0.16	0.44	1.13	0.69	24.25
Carbohydrate/Sugar Ratio	2.46 ± 0.74	1.09	3.82	2.73	30.2
Dry weight leaf	27.63 ± 6.21	17.39	41.48	24.09	22.48
Dry weight shoot	10.32 ± 2.32	7.6	15.04	7.44	22.44
Dry weight root	10.9 ± 4.07	3.42	18.37	14.95	37.32
Dry weight total	48.84 ± 11.05	29.47	71.25	41.78	22.62
Fresh weight leaf	112.46 ± 25.28	70.79	168.83	98.04	22.48
Fresh weight shoot	51.62 ± 11.33	37.82	74.76	36.94	21.94
Fresh weight root	44.4 ± 16.56	13.96	74.79	60.83	37.3
Fresh weight total	214.73 ± 59.42	126.9	386.31	259.41	27.67
Lenght root	46.81 ± 12.05	32	73	41	25.74
Volume root	43.67 ± 12.93	20	60	40	29.6

### Morphological attributes

The results of the analysis of variance showed that plant height, leaf number, leaf area, root volume, root length, chlorophyll index, and fresh and dry weight were significantly affected by foliar spraying of Gibberellic acid and benzyladenine compared with control.

### Plant height and leaf number

The comparison of means showed that plant height and leaf number were significantly affected by plant growth regulators. Gibberellic acid at 200 mg/l and gibberellic acid + benzyladenine at 200 mg/l concentrations were the most effective treatments and increased plant height and leaf number by 32.92 and 62.76 folds compared with the control (Table 2).

**Table 2:** Effect of GA_3_ and BA on plant growth parameters of dwarf schefflera.

Treatment	Internode (mm)	Root length (cm)	Root volume (m^3^)	Leaf area (cm^2^)	No. of leaves/plant	Plant height (cm)	Diameter stem (mm)	Leaf chlorophyllIndex
NO PGR	40.83 ± 1.16 cd	34.25 ± 1.25 f.	20 ± 0.05 d	70.63 ± 2.53 c	23.5 ± 0.47 e	82 ± 2.3 d	1.21 ± 0.03 a	46.07 ± 1.25 d
100 mg/L GA_3_	48.58 ± 1.1abc	41.50 ± 1 de	51.50 ± 1.05 ab	72.72 ± 2.62 c	28 ± 1.01 cd	93.75 ± 2.01 c	0.98 ± 0.03 b	49.78 ± 1.06 cd
200 mg/L GA_3_	44.2 ± 1.58abc	44 ± 1 de	60 ± 2.3 a	83.26 ± 2.44 abc	32.25 ± 0.57 bc	90.75 ± 1.033 cd	0.9 ± 0.01 b	53.04 ± 1.28 bc
100 mg/L BA	52.99 ± 1.22ab	34.50 ± 1.5 f.	32.5 ± 1.5 c	74.66 ± 2.35 bc	27.5 ± 0.94 d	92 ± 2.3 cd	0.84 ± 0.05 b	50.95 ± 1.95 bcd
100 mg/L GA_3_ + 100 mg/L BA	35.99 ± 1.96 c	38.75 ± 1.25 ef	33 ± 1 c	77.72 ± 1.16 abc	29.75 ± 0.62 bcd	96.75 ± 1.02 c	0.83 ± 0.07 b	52.58 ± 3.45 bc
200 mg/L GA_3_ + 100 mg/L BA	42.24 ± 1.4 cd	46.50 ± 1.75 cd	51 ± 1.5 ab	86.91 ± 2.37 ab	33.75 ± 0.85 ab	97.75 ± 1.39 bc	0.83 ± 0.02 b	57.08 ± 0.98 ab
200 mg/L BA	56.58 ± 1.87 a	51 ± 1.5 c	42 ± 1 bc	81.23 ± 2.15 abc	28.75 ± 0.79 cd	109 ± 2.61 a	0.81 ± 0.07 b	53.46 ± 2.96 bc
100 mg/L GA_3_ + 200 mg/L BA	44.33 ± 1.64 abc	59 ± 1.5 b	49 ± 1.9 ab	81.05 ± 2.83 abc	30.25 ± 1.17 bcd	97 ± 1.78 c	0.78 ± 0.09 b	54.03 ± 2.219 abc
200 mg/L GA_3_ + 200 mg/L BA	43.33 ± 2.07 abc	71.75 ± 2 a	54 ± 1 a	89.19 ± 2.71 a	38.25 ± 1.25 a	107.75 ± 1.51 ab	0.76 ± 0.008 b	59.99 ± 1.41 a

† Means in each column with the same letters are not statistically significantly different at p < 0.05 based on Duncan's multiple range test.

### Leaf area

Leaf area was significantly higher in all treatments compared with control, and the highest increase was observed for Gibberellic acid at 200 mg/l with 89.19 cm^2^. According to the results, leaf area significantly increased with increasing growth regulator concentration (Table 2).

### Root volume and length

All treatments significantly increased root volume and length compared with control. The highest effect belonged to the gibberellic acid + benzyladenine combination, which doubled root volume and length compared with the control (Table 2).

### Stem diameter and internode length

The highest values for stem diameter and internode length were observed in control and gibberellic acid + benzyladenine at 200 mg/l treatments, respectively.

### Chlorophyll index

The chlorophyll index was improved by all treatments compared with the control. The highest value for this trait was observed in gibberellic acid + benzyladenine at 200 mg/l treatment, which was 30.21% higher than that of the control (Table 2).

### Measurement of physiological attributes

The results showed that the treatments led to significant differences in pigment content, reducing sugars, and soluble carbohydrates.

### Plant pigments (chlorophylls and carotenoids)

Gibberellic acid + benzyladenine treatment resulted in the highest photosynthetic pigment content. This trait in all treatments was significantly higher than control.

The results showed that all treatments led to improved chlorophyll content in dwarf schefflera. However, the highest value for this trait was observed in gibberellic acid + benzyladenine treatment, which was 36.95% higher than that of the control (Table 3).

**Table 3:** Effect of GA_3_ and BA on the photosynthetic pigment of dwarf schefflera.

Photosynthetic pigment (mg.ml-1)					
	Ch. a	Ch. b	Ch. total	Carotenoid	Yield pigment
NO PGR	15.94 ± 0.56 bc	5.42 ± 0.18 c	21.36 ± 0.7 c	3.42 ± 0.17 a	24.78 ± 0.64 b
100 mg/L GA3	16.24 ± 0.16 bc	8.06 ± 0.53 ab	24.3 ± 0.69 bc	3.55 ± 0.33 a	27.86 ± 0.96 ab
200 mg/L GA3	21.83 ± 0.85 a	7.34 ± 0.57 abc	29.18 ± 0.61 ab	2.66 ± 0.24 abc	31.84 ± 0.66 a
100 mg/L BA	14.76 ± 0.36 c	5.72 ± 0.35 c	20.49 ± 0.66 c	3.10 ± 0.11 ab	23.6 ± 0.25 b
100 mg/L GA3 + 100 mg/L BA	19.02 ± 1.3 ab	6.48 ± 0.53 bc	25.5 ± 1.79 abc	3.14 ± 0.12 ab	28.64 ± 0.71 ab
200 mg/L GA3 + 100 mg/L BA	21.16 ± 0.71 a	8.61 ± 0.5 ab	29.77 ± 1.19 ab	2.72 ± 0.17 abc	32.5 ± 1.02 a
200 mg/L BA	18.54 ± 1.01 abc	6.46 ± 0.13 bc	25 ± 1.14 abc	3.03 ± 0.14 ab	28.03 ± 1.2 ab
100 mg/L GA3 + 200 mg/L BA	21.73 ± 2.1 a	9.06 ± 1.6 a	30.79 ± 1.62 a	2.07 ± 0.58 c	32.31 ± 1.58 a
200 mg/L GA3 + 200 mg/L BA	21.25 ± 0.09 a	8.15 ± 0.57 ab	29.4 ± 0.62 ab	2.35 ± 0.24 bc	31.76 ± 0.37 a

† Means in each column with the same letters are not statistically significantly different at p < 0.05 based on Duncan's multiple range test.

Results related to Chlorophyll b were entirely similar to those of chlorophyll a, with the only difference being the extent of increase in this pigment, which was 67.15% higher than the control (Table 3).

The treatments led to a significant increase in total chlorophyll compared with control. 200 mg/l gibberellic acid + 100 mg/l benzyladenine treatment led to the highest value for this trait, which was 50% higher than the control (Table 3).According to the results, control and 100 mg/l benzyladenine led to the highest value for carotenoid in dwarf schefflera (Table 3).

**Figure 1:**
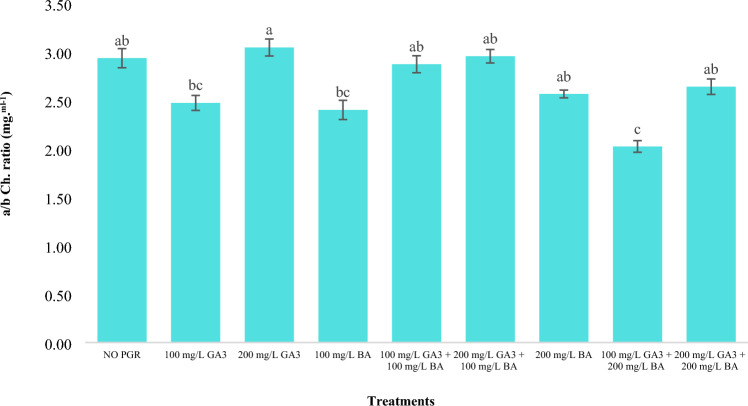
Effects of gibberellic acid and benzyladenine on a/b Ch. Ratio of Dwarf schefflera*.* (GA_3_: gibberellic acid and BA: benzyladenine). In each figure means with same letters had no significant difference with each other (P < 0.01).

The results showed that the treatments led to a significant increase in chlorophyll a to chlorophyll b at 200 mg/l gibberellic acid (Fig. 1).

### Fresh and dry weight

The effect of all treatments on the fresh and dry weight of dwarf schefflera was significantly higher than the control. Gibberellic acid + benzyladenine at 200 mg/l was the most effective treatment and increased the fresh weight by 138.45% compared with the control. All treatments significantly improved the plant dry weight compared with control except for 100 mg/l benzyladenine, and gibberellic acid + benzyladenine at 200 mg/l led to the highest value for this trait (Table 4).

**Table 4:** Effect of GA_3_ and BA on fresh weight and dry weight of leaf, stem, root and the total weight of dwarf schefflera.

	Fresh weight (g)				Dry weight (g)			
	Leaf	Shoot	Root	Total	Leaf	Shoot	Root	Total
NO PGR	77.02 ± d	37.89 ± g	23.89 ± b	138.8 ± d	18.92 ± 0.47d	7.61 ± 0.01 g	5.86 ± 0.44 b	32.39 ± 0.92 d
100 mg/L GA_3_	77.88 ± d	45.45 ± ef	24.38 ± b	147.68 ± cd	19.13 ± 0.17d	8.64 ± 0.38 f.	5.98 ± 0.39 b	33.75 ± 1.18 d
200 mg/L GA_3_	97.18 ± cd	43.05 ± f	60.98 ± a	216.56 ± cd	29.17 ± 0.67 bc	11.35 ± 0.18 c	13.53 ± 0.43 a	54.06 ± 1.42 b
100 mg/L BA	113.87 ± bc	43.53 ± f	33.07 ± ab	190.48 ± bcd	27.97 ± 0.55 bc	8.72 ± 0.03 f.	8.12 ± 0.99 ab	44.73 ± 1.6 c
100 mg/L GA_3_ + 100 mg/L BA	122.28 ± b	51.48 ± d	44.91 ± ab	218.67 ± bc	24.03 ± 0.53 cd	8.66 ± 0.24 f.	14.97 ± 0.15 a	47.66 ± 1.43 bc
200 mg/L GA_3_ + 100 mg/L BA	133.7 ± ab	65.51 ± b	42.09 ± ab	241.3 ± b	32.48 ± 0.46 ab	13.17 ± 0.26 b	10.33 ± 0.25 ab	56.35 ± 1.45 b
200 mg/L BA	116.3 ± bc	47.7 ± e	53.77 ± a	217.77 ± bcd	28.57 ± 0.37 bc	9.59 ± 0.23 c	13.2 ± 0.58 a	51.37 ± 1.44 bc
100 mg/L GA_3_ + 200 mg/L BA	118.75 ± bc	56.43 ± c	55.11 ± a	230.30 ± b	30.03 ± 0.22 b	10.35 ± 0.11 d	11.03 ± 0.69 ab	51.42 ± 0.35 bc
200 mg/L GA_3_ + 200 mg/L BA	154.48 ± a	73.55 ± a	61.36 ± a	289.39 ± a	37.95 ± 0.74 a	14.79 ± 0.14 a	15.07 ± 0.13 a	67.81 ± 0.73 a

† Means in each column with the same letters are not statistically significantly different at p < 0.05 based on Duncan's multiple range test.

### Soluble carbohydrates to reducing sugars ratio

**Figure 2:**
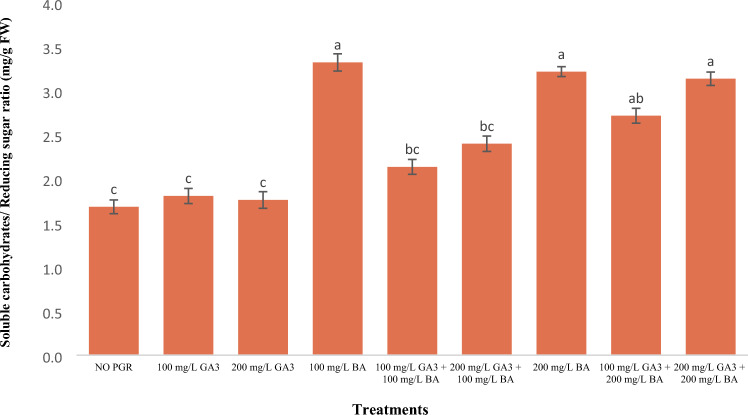
Effects of: gibberellic acid and benzyladenine on soluble carbohydrates/reducing sugar ratio of Dwarf schefflera*.* (GA_3_: gibberellic acid and BA: benzyladenine). In each figure means with same letters had no significant difference with each other (P < 0.01).

Most treatments had a significant difference with the control in terms of this trait, with the highest value belonging to 100 and 200 mg/l benzyladenine and 200 mg/l gibberellic acid + benzyladenine (Fig. 2).

A stepwise regression analysis was performed to determine the effective attributes and gain a better understanding of the relationship between the independent variables and the number of leaves in dwarf schefflera. Root volume was the first variable to enter the model and explained 44% of variations. The next variable was root fresh weight and these two variables explained 63% of variations in the number of leaves (Table 5).

**Table 5:** Stepwise regression for leaf number Dwarf schefflera.

Variables entered to model	Step	
	**1**	**2**
Intercept	38.39	27.56
Fresh weight of root	−	3.71
Root volume	− 21.33	− 20.43
R^2^ (%)	44	63

Significant values in bold.

The result of the stepwise regression analysis was as follows:

$$Y=27.56+3.71{X}_{1}-20.43{X}_{2}$$

In which Y is leaf number, X1 is root dry weight and X2 is root volume.

**Figure 3:**
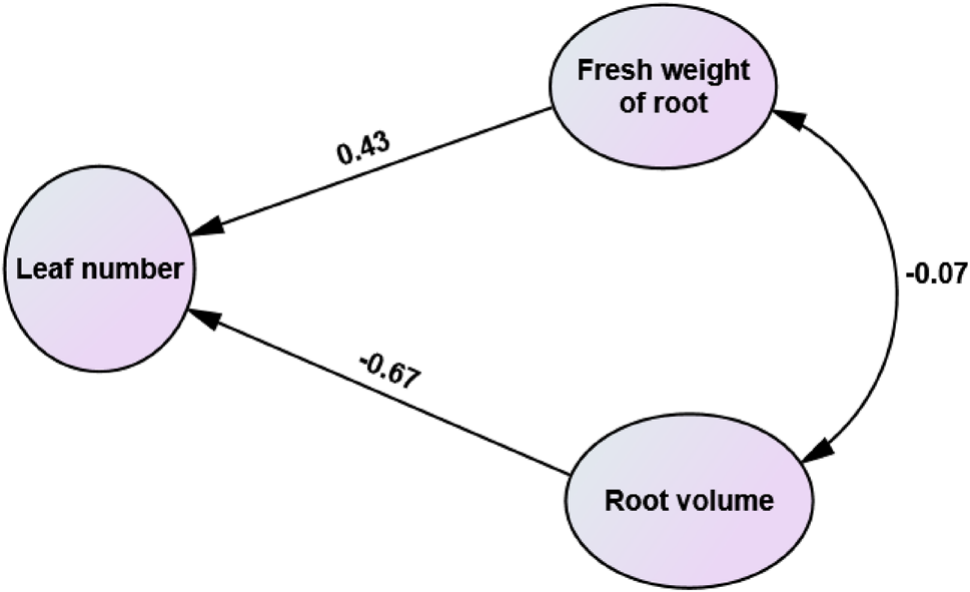
Path analysis of studied traits on leaf number in dwarf schefflera.

Path analysis was performed for better interpretation of the stepwise regression (Table 6 and Fig. 3). The highest positive effect on leaf number is related to root fresh weight (0.43) which correlated positively with leaf number (0.47). This indicates that this trait directly affects yield and its indirect effect through other traits is negligible and this trait can be used as a selection criterion in dwarf schefflera breeding programs. The direct effect of root volume was negative (− 0.67). The effect of this trait on leaf number was direct and its indirect effect was negligible. This shows that the higher the root volume, the lower the leaf number.

**Table 6:** Direct (diagonal values) and indirect (values outside diagonal) effects of studied traits on leaf number.

Trait	Root fresh weight	Root volume	Correlation with leaf number
Root fresh weight	0.43	0.04	0.47
Root volume	− 0.03	− 0.64	− 0.67

**Figure 4:**
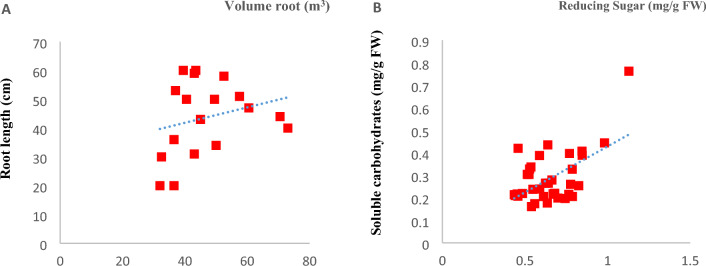
Results of regression linear root length (**A**) and soluble carbohydrates (**B**) in Dwarf shefflera.

Figure 4 shows the changes in the linear regression for root volume and reducing sugars. Based on the regression coefficient, each unit of change in root length and soluble carbohydrates means 0.6019 and 0.311 units of change in root volume and reducing sugars.

**Figure 5:**
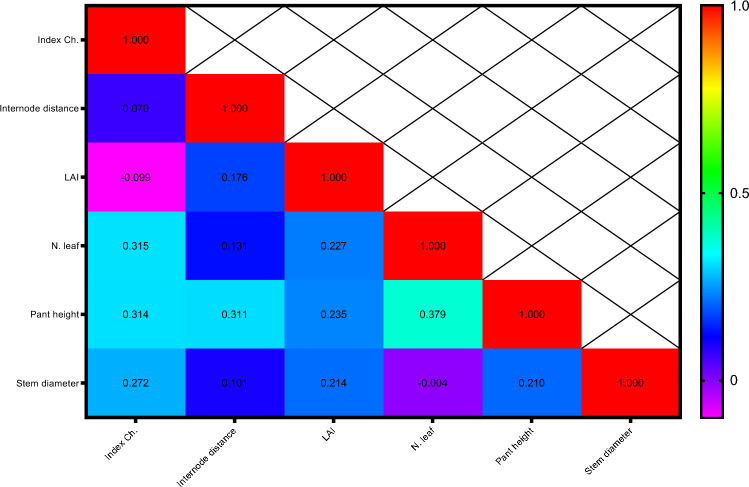
Heat map of mutual relations of variables in correlation coefficient for growth indicators. # Y axis are respectively: 1- Index Ch., 2- Internode distance, 3- LAI, 4- N. leaf, 5-Pant height, 6- Stem diameter. # X axis are respectively: A- Index Ch., B- Internode distance, C- LAI, D- N. leaf, E-Pant height, F- Stem diameter.

Pearson's correlation coefficients for growth attributes are presented in Fig. 5. The results showed that leaf number and plant height (0.379*) had the highest positive and significant correlation.

**Figure 6:**
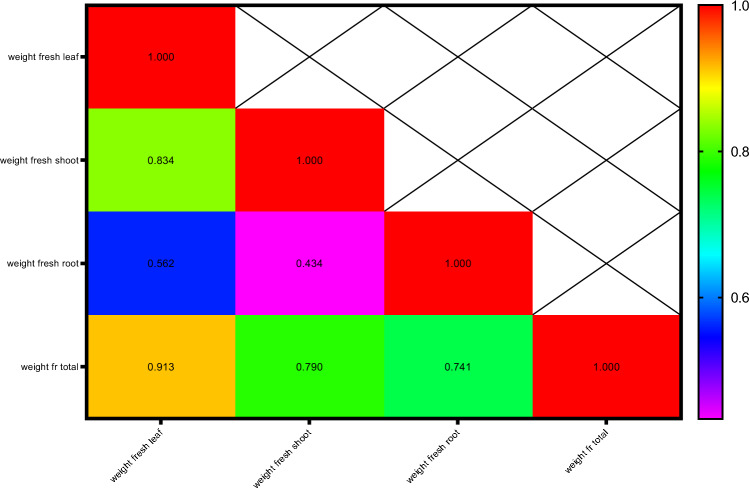
Heat map of mutual relations of variables in correlation coefficient for fresh weight. # Y axis are respectively: 1- Weight fresh leaf, 2- Weight fresh shoot, 3-Wweight fresh root, 4-Weight fresh total. # X axis are respectively: A- Weight fresh leaf, B- Weight fresh shoot, C-Wweight fresh root, D-Weight fresh total.

Pearson's correlation coefficients for fresh weight-related attributes are presented in Fig. 6. The results showed that leaf fresh weight with shoot dry weight (0.834**), total dry weight (0.913**) and root dry weight (0.562*); and total dry weight with shoot dry weight (0.790**) and root dry weight (0.741**) had the highest positive and significant correlations.

**Figure 7:**
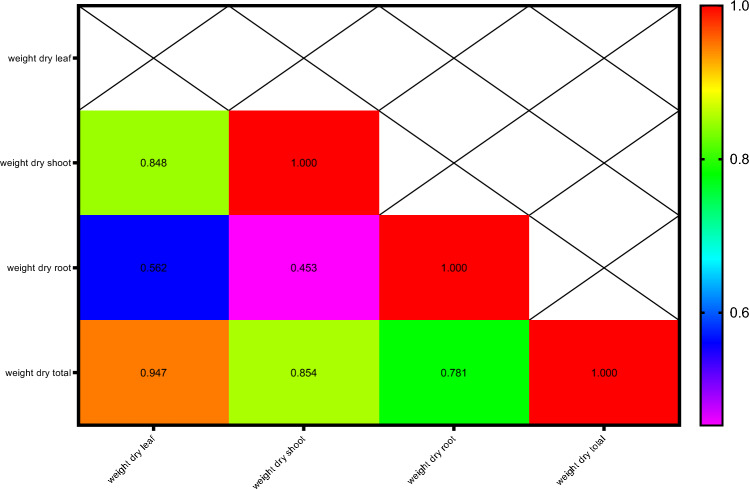
Heat map of mutual relations of variables in correlation coefficient for dry weight. # Y axis are respectively: 1- Weight dry leaf, 2- Weight dry shoot,3-Wweight dry root, 4-Weight dry total. # X axis are respectively: A- Weight dry leaf, B- Weight dry shoot,C-Wweight dry root, D-Weight dry total.

Pearson's correlation coefficients for dry weight- related attributes are presented in Fig. 7. The results showed that leaf dry weight with shoot dry weight (0.848**) and total dry weight (0.947**);total dry weight with shoot dry weight (0.854**) and total dry weight (0.781**) had the highest positive and significant correlations.

**Figure 8:**
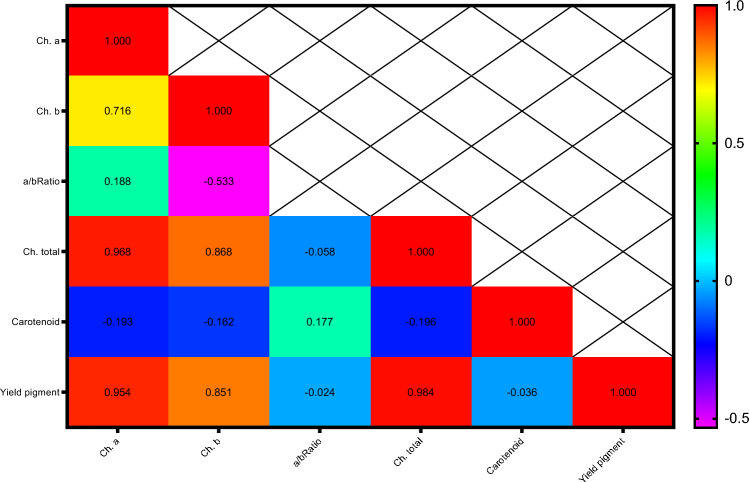
Heat map of mutual relations of variables in correlation coefficient for chlorophyll content. # Y axis are respectively: 1- Ch. a, 2- Ch. b,3- a/b Ratio, 4-Ch. total, 5- Carotenoid, 6- Yield pigment. # X axis are respectively: A- Ch. a, B- Ch. b,C- a/b Ratio, D-Ch. total, E- Carotenoid, F- Yield pigment.

Pearson's correlation coefficients for pigment attributes are presented in Fig. 8. The results showed that chlorophyll a with chlorophyll b (0.716**), total chlorophyll (0.968**) and total pigments (0.954**); chlorophyll b with total chlorophyll (0.868**) and total pigments (0.851**); and total chlorophyll with total pigments (0.984**) had the highest positive and significant correlations.

## Discussion

Dwarf schefflera is a popular indoor plant worldwide, and its growth and development are currently receiving a lot of attention. The use of plant growth regulators has resulted in significant differences, with all treatments improving plant height compared to the control group. While plant height is generally controlled by genetics, studies have shown that the application of plant growth regulators can increase or decrease plant height. The highest plant height and leaf number were observed in the treatment that used gibberellic acid + benzyladenine at 200 mg/l, reaching 109 cm and 38.25, respectively. Similar plant height increases due to gibberellic acid treatment have been observed in pot marigold, leucanthemum^21^, daylilies^22^, daylilies, false aralia, and peace lily, according to previous studies (SalehiSardoei et al.^52^) and peace lily^23^.

**Figure 9:**
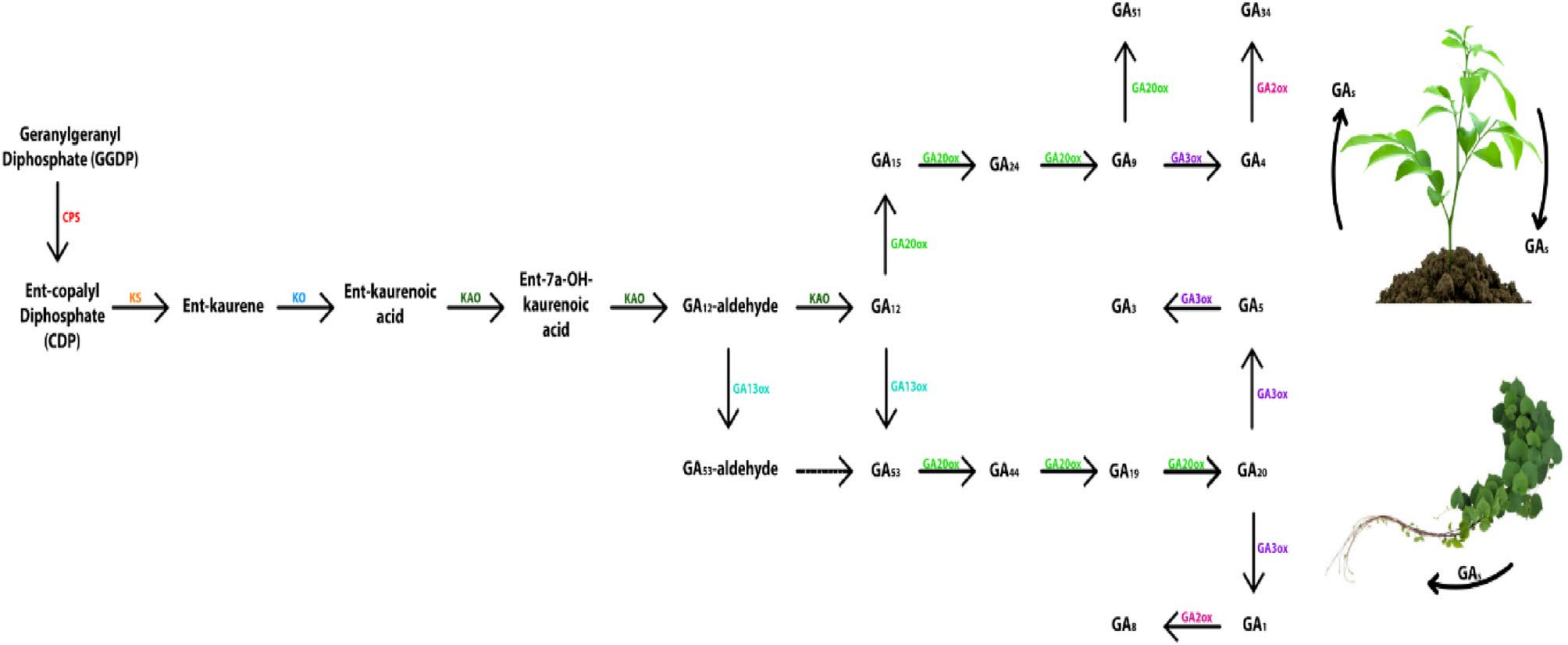
Biosynthesis of GA and levels of endogenous bioactive GA in plants; a schematic representation of a plant (right) and GA biosynthesis (left). The arrows are color-coded to correspond to the GA forms indicated in the biosynthetic pathway; red arrows represent reduced GA levels due to plant organ positioning, while black arrows indicate increased GA levels. In several plants such as rice and watermelon, the GA content in the leaf base or its lower part is high^30^. Furthermore, several reports have revealed that the content of bioactive GA has decreased following the elongation from the base to the leaf^34^. The precise level of gibberellins has not been evident in these cases.

Gibberellic acids (GAs) play an important role in various physiological processes in plants. They stimulate cell division, cell elongation, stem elongation, and size increment^24^. GAs induce cell division and elongation in the shoot apex and meristematic tissue^25^. Leaves also exhibit changes such as reduced stem thickness, smaller leaf size, and brighter green coloration^26^. Studies utilizing inhibitory or stimulatory factors have shown that calcium ions, derived from internal sources, act as secondary messengers in the signal transduction pathways of gibberellins within *Sorghum corolla*^27^. GAs increase plant length by stimulating the synthesis of enzymes that induce cell wall loosening, such as XET or XTH, Expansins, and PME^28^. This leads to cell enlargement due to cell wall loosening and water entry into the cell^29^. Application of GA_7_, GA_3_, and GA_4_ increases stem elongation^30,31^. Gibberellic acid causes stem elongation in dwarf plants, while in rosette plants, GAs delay leaf growth and internode elongation^32^. However, just before the reproductive stage, stem lengths increase by 4 to 5 times more than the initial height^33^.The biosynthesis process of GA in plants is summarized in Fig. 9.

Leaf number and area were also significantly affected by plant growth regulators. The results showed that an increased concentration of plant growth regulators led to a significant increase in leaf area and number. It was also reported that benzyladenine increased leaf number in calla lily^15^. According to the findings of this study, all treatments improved leaf area and number. Gibberellic acid + benzyladenine was the most effective treatment and led to the highest number of leaf number and leaf area. The increased number of leaves in instances where dwarf schefflera is grown indoors can be notable.

Internode length increased by GA_3_ application in comparison withbenzyladenine (BA)or hormone-free treatments. This result Is logical due to the role of GA in growth improvement^7^. Stem growth showed similar results as well. Gibberellic acid increased stem length but decreased stem diameter. Nonetheless, the combined application of BA and GA_3_ remarkably increased stem length. This increase was higher compared with the plants treated with BA or hormone-free ones. Although gibberellic acid and cytokinin (CK) usually improve plant growth, they show contrasting effects on different processes in some occasions^35^. For instance, there is a negative interaction in hypocotyl length increase in plants treated with GA and BA^36^. On the other hand, BA significantly increased root volume (Table 1). An increase in root volume due to exogenous BA is reported in numerous plants such as dendrobium and epipremnum^37,38^.

All hormonal treatments increased the number of new leaves. The natural increase in leaf area and stem length by the combined treatment is commercially desirable. The number of new leaves is an important index for vegetative growth. The exogenous application of hormones has not been used for the commercial production of dwarf schefflera. However, growth-enhancing effects of GA and CK application in a balanced manner may provide new insights to improve the cultivation of this plant. It may be noted that the synergistic effect of BA + GA_3_ treatment was higher than the sole application of GA or BA. Gibberellic acid increased the number of new leaves. Since new leaves are developing, an increased number of new leaves may restrict leaf growth^39^. It is reported that GA improved sucrose translocation from sink to source organs^40,41^. Also, the exogenous application of GA in perennial plants may prevent the transition from vegetative to reproductive growth by increased growth of vegetative organs such as leaves and roots^42^.

The effect of GA on increased plant dry matter may be attributed to the enhancement of photosynthesis via increased leaf area^43^. It is reported that GA led to increased leaf area in Maize^34^. The results show that an increased concentration of BA to 200 mg/l improved the auxiliary branch length and number as well as root volume.Gibberellic acid affects cell processes such as stimulation of cell division and elongation and thus, improves vegetative growth^43^. Also, GAs decrease cell water potential by cell wall expansion through starch hydrolysis to sugar, leading to water entry into cells and eventually, cell elongation^44^.

Plant growth regulators directly affect photosynthesis through chlorophyll hydrolysis and biosynthesis^45^. Plant growth regulators also prevent chlorophyll degradation and enhance amino acid uptake and protein maintenance in plants^46^. Improved content of photosynthetic pigments is reflected in photosynthesis and increases carbohydrate content^47^. Application of plant growth regulators increased the content of photosynthetic pigments in dwarf schefflera, and GA + BA at 200 mg/l had the highest effect in this regard. It is reported that BA application improves photosynthetic pigments in peace lily^3^. Moreover, GA and BA increased chlorophyll content in calla lily^15^. According to the findings of the present study, the chlorophyll index increased with increasing plant growth regulator concentration, and GA had a greater effect in this regard compared with BA. Gibberellic acid stimulates sucrose synthesis and its translocation from leaves to phloem^48^. Sucrose synthesis stimulation and its translocation to phloem by GA application not onlyimprove shoot growth which serves as shoots, but another part of sucrose is translocated to subterranean organs, improving root growth^49^. On the other hand, GAs stimulate the activation of enzymes such as proteases and this, converts proteins to amino acids including tryptophan, which is a precursor for auxin^50^. Therefore, they exert some of their effects indirectly through auxin as well^26^. The results related to leaf chlorophyll showed that GA significantly differed from control, which was in accordance with the results obtained for freesia^51^ and Aralia^52^. Gibberellic acid has structural roles in the chloroplast membrane and stimulates photosynthesis^53^. Studies conducted on plant growth regulators such as GA show that they can improve the content of important pigments including carotenoids^54,55^. Chlorophyll is essential to plants regarding interception and utilization of solar energy during photosynthesis. Therefore, the effect of plant growth regulators on chlorophyll hydrolysis and biosynthesis directly impacts photosynthesis ^56^.In general, gibberellins, cytokinins, and auxins delay chlorophyll breakdown, while ethylene and abscisic acid promote it in leaves^57^. Gibberellins prevent chlorophyll and nitrogen breakdown during the senescence process, possibly due to the structural role of gibberellins in the chloroplast membrane as well as stimulation of photosynthesis^58^. Chlorophyll breakdown is an evident sign of leaf senescence, while plant growth regulators affect leaf senescence^59^. Leaf senescence correlates closely with lipid peroxidation and the activity levels of antioxidants, catalase (CAT), ascorbate peroxidase (APX), peroxidase (POD), and superoxide dismutase (SOD)^60,61^. During senescence process, leaf protein content decreases^62^, and the level of free radicals’ activity (ROS) increases Martins et al. ^1^. Protein and unsaturated fatty acid peroxidation lead to DNA damage, causing cell damage and eventual cell death^63^. As antioxidants counteract free radical activity, antioxidant activity decreases, and ROS levels increase during senescence process^63,64^. On the other hand, cytokinins delay leaf senescence by regulating antioxidant activity^65^. A significant portion of cytokinins is found in plastids^66^ and the primary goal of cytokinins is the growth and development of plastids^67^. Evidence suggests that the presence of active cytokinins stimulates the structural growth of chloroplasts and photosynthesis and synthesis of photosynthetic pigments^68,69^.

Plant dry weight was significantly affected by GA + BA at 200 mg/l concentration. This increase may be attributed to an increase in plant height, and the number of leaves and auxiliary branches. It was observed that other parameters including leaf area, chlorophyll and carotenoid contents, and reducing sugars and soluble carbohydrate contents also improved. These parameters can improve plant dry weight. In accordance with the present study, the application of plant growth regulators such as GA and BA increased leaf dry weight compared with the control^70^. The positive effect of GA on plant dry weight may be attributed to an improvement in photosynthesis via increased leaf area^71^. Also, results of a study on aralia show that plant growth regulators increase plant fresh and dry weights^72^. Plant growth regulators will not be completely mobile if they are not sprayed directly on the leaf attached to the plant^73^. If only a single treated leaf remains green while other leaves abscise, assimilates will preferably translocate to the tissues treated with plant growth regulators and accumulate^74^. It is hypothesized that the hormone leads to assimilate translocation by establishing a new source-sink relationship^41^. The increase in fresh and dry weight of dwarf schefflera by cytokinins may be due to increased cell division and elongation. The fresh weight of leaves, shoots, roots and total fresh weight of dwarf schefflera significantly increased by application of GA + BA at 200 mg/l concentration. The increase in plant fresh weight may be due to an increased number of leaves per plant. Similar results are reported by Bhattacharya et al.^75^ in geranium. Inadequate translocation of photosynthetic assimilates to growing organs is currently the most important limiting factor for plant production in many species^76^. This limitation may be overcome using synthetic growth regulators which improve canopy structure and production by manipulating the source-sink relationship^77^. In all treatments, plant fresh and dry weights increased compared with control.

Reducing sugars and soluble carbohydrates were also affected by plant growth regulators GA and BA. Reducing sugars increased by 20.18% compared with control in GA + BA 200 mg/l treatment. Application of GA and BA increased soluble sugars in Jerusalem cherry^78^. The results of the present study confirm these reports. Sugar content (soluble solids) is an important factor in the determination of growth. Thus, the higher the percentage of reserved carbohydrates, the greater the growth traits^78,79^. A combination of biochemical and physiological factors along with the environmental effects determine the plant yield^80^. According to the present study, there was a great difference among the treatments regarding reducing sugar and soluble carbohydrate contents. Correlation among plant growth regulators GA and BA and increased content of reducing sugars and soluble carbohydrates is reported in false aralia leaves^52^. Plant growth regulators GA and BA accelerate the synthesis of photosynthetic proteins and result in cell development in some tissues and organs. This will lead to increased content of soluble solids and carbohydrates, and translocation of assimilates from leaf to buds which results in increased cell turgor and growth^36,38,45,81^.Facilitation of growth by GA is because of the increased demand for soluble carbohydrates due to increased invertase expression^82^. Hormones like gibberellins affect invertase gene expression. Hexoses produced in target tissues by invertase activity not only serve as a carbon and energy source for plant growth, but also provide the force and energy necessary for cell elongation through reducing cell osmotic potential as well as increasing water absorption^83^. Gibberellic acid strengthens the chloroplast structure, stimulates photosynthesis, and maintains chlorophyll stability^84,85^. It appears that gibberellic acid enhances starch hydrolysis into sucrose and fructose, in turn reinforcing cell walls and increasing the hydrocarbon compounds contents, consequently increasing the soluble solid content (SSC)^86,87^.

Coefficient of variation and linear regression are used to determine the relationship of linear changes in two variables, and are very important in plant breeding studies^88^. SalehiSardoei et al.^89^ reported that there is a significant and positive correlation between ion leakage and water core in citrus cultivars. Correlation among attributes may help plant breeding researchers to indirectly select important stress traits through other traits that are easier to measure^90^. Although correlation coefficients of physiological traits are useful for determining stress-related attributes, they fail to correctly describe the relationship among traits. Therefore, it is essential to determine the direct and indirect effects of these traits in plant breeding programs^88^. Stepwise regression may be used to select salient variables out of numerous attributes measured in order to determine the greatest share in the explanation of tolerance^91^. Stepwise regression can eliminate ineffective traits in the model and select traits which explain a considerable part of the variation^88,91^. Since the present study contained numerous traits which correlated significantly and positively with leaf number, it was feasible to further investigate these traits using other statistical methods to determine important yield-affecting attributes. Thus, path analysis was used to obtain further information about the relationship between the studied traits and their effect on leaf number of dwarf schefflera.

Stepwise regression was performed to determine the most effective traits on leaf number, root volume and root fresh weight and elimination of negligible traits in order to start the path analysis. Root fresh weight with R^2^ = 44% and root volume with R^2^ = 63% entered the model. In general, based on the stepwise regression results, the first and second variables to enter the model were root fresh weight and root volume, which had the greatest share in the explanation of leaf number.

The obtained results from simple correlation and stepwise regression were interpreted better by subjecting the variables that entered the model to path analysis. All direct path coefficients were found to be significant. Root fresh weight had the greatest positive effect on leaf number (0.43), and it was positively correlated with leaf number (0.47). This indicates that root fresh weight directly affects yield, and its indirect effects through other traits are negligible. Therefore, this trait can be used as a selective criterion for dwarf Schefflera breeding programs. Root volume and root fresh weight had the highest positive and significant effect on other traits.

## Conclusion

This study aimed to examine the impact of external application of BA and GA3 on the growth of dwarf Schefflera plants. The research revealed that the use of plant growth regulators helps in cell division and differentiation, leading to better growth and development. Since shoot and leaf growth is essential for houseplants, plant growth regulators can be applied to enhance their appearance and growth, particularly when used in combination due to their more cost-effective nature. The study found that the most effective treatment was the application of GA + BA at a dosage of 200 mg/l.

## Data Availability

All the data generated/ analyzed during the study are available with the corresponding author on reasonable request.
